# Development and validation of a nomogram to predict postoperative delirium in type B aortic dissection patients underwent thoracic endovascular aortic repair

**DOI:** 10.3389/fsurg.2022.986185

**Published:** 2022-09-08

**Authors:** Wanbing Huang, Qiansheng Wu, Yufen Zhang, Chong Tian, Haishan Huang, Hui Wang, Jing Mao

**Affiliations:** ^1^ Department of Nursing, Tongji Hospital, Tongji Medical College, Huazhong University of Science and Technology, Wuhan, China; ^2^ School of Nursing, Tongji Medical College, Huazhong University of Science and Technology, Wuhan, China; ^3^ Division of Cardiothoracic and Vascular Surgery, Tongji Hospital, Tongji Medical College, Huazhong University of Science and Technology, Wuhan, China

**Keywords:** nomogram, prediction model, postoperative delirium, type B aortic dissection, thoracic endovascular aortic repair

## Abstract

**Objective:**

Postoperative delirium (POD) is a common postoperative complication after cardiovascular surgery with adverse outcomes. No prediction tools are currently available for assessing POD in the type B aortic dissection (TBAD) population. The purposes of this study were to develop and validate a nomogram for predicting POD among TBAD patients who underwent thoracic endovascular aortic repair (TEVAR).

**Methods:**

The retrospective cohort included 631 eligible TBAD patients who underwent TEVAR from January 2019 to July 2021. 434 patients included before 2021 were in the develop set; 197 others were in the independent validation set. Least absolute shrinkage and selection operator (LASSO) and logistic regression were applied to identify the most useful predictive variables for constructing the nomogram. Discrimination and the agreement of the model was assessed with the area under the receiver operating characteristic curve (AUC), Brier score and the Hosmer-Lemeshow goodness-of-fit test. The results were validated using a bootstrap resampling and the validation set.

**Results:**

The incidence rate of POD observed in the development and validation cohort were 15.0% and 14.2%, respectively. Seven independent risk factors, including age ≥60 years, syncope or coma, postoperative blood transfusion, atelectasis, estimated glomerular filtration rate (eGFR) <80 ml/min/1.73 m^2^, albumin <30 g/L, and neutrophil to lymphocyte ratio, were included in the nomogram. The model showed a good discrimination with an AUC of 0.819 (95% CI, 0.762–0.876) in the developed set, and adjusted to 0.797 (95% CI, 0.735–0.849) and 0.791 (95% CI, 0.700–0.881) in the internal validation set and the external validation, respectively. Favorable calibration of the nomogram was confirmed in both the development and validation cohorts.

**Conclusion:**

The nomogram based on seven readily available predictors has sufficient validity to identify POD risk in this population. This tool may facilitate targeted initiation of POD preventive intervention for healthcare providers.

## Introduction

Aortic dissection (AD) is a rare and life-threatening cardiovascular disease with high mortality in which blood passing into the media layer due to an aortic intimal tearing (1, 2). According to the Stanford classification system, type B AD (TBAD) does not involve the ascending aorta and has a much better prognosis than type A AD (TAAD) (3, 4). With the development and improvement of medical techniques over the past decade, thoracic endovascular aortic repair (TEVAR) has emerged as a more attractive choice for TBAD due to its minimally invasive feature compared with conventional open surgery (2, 5). Evidence has shown that timely TEVAR can significantly reduce mortality and improve the overall prognosis of TBAD patients (6). However, multiple postoperative complications may still occur after endovascular procedures with relatively high incidence, which are known to affect patient clinical outcomes and quality of life (2, 7). Studies concerning early risk identification and reduction of these complications are of great significance.

Postoperative delirium (POD) is a neuropsychiatric disorder characterized by an acute onset of fluctuating changes in mental status with impaired consciousness and attention deficits (8). Reportedly, it is a common postoperative complication after cardiac or vascular surgery, with an incidence of 29.8% (9) to 39% (10). However, studies on POD in patients with TBAD are very limited. In a recent retrospective study among TBAD patients who underwent TEVAR, the incidence of POD was 13.3% (11). Additionally, POD has been found to be associated with increased severe complications, such as stroke and spinal cord ischemia, and greater in-hospital and follow-up mortality (11). Furthermore, AD patients with POD had significantly longer hospitalization and ICU stays and higher hospital costs (11, 12). At the same time, patients with delirium require an average of 60 min of additional care, significantly increasing the workload of clinical nurses (13). All of these would place an enormous burden on individuals, society and the healthcare system.

Noteworthily, there has been strong evidence that early applied multifactorial nonpharmacologic interventions for high-risk patients can reduce the odds of delirium by 44% (14). Thus, the accurate identification of risk factors and effective prediction of POD are extremely important. Several investigations on POD risk factors have already been reported, and some factors have been revealed to be strongly related to the development of delirium, such as advanced age, renal dysfunction, and inflammation (9, 12, 15, 16). Nevertheless, no prediction tools are currently available for assessing delirium in the TBAD population. Nomogram is a tool that has been useful in accurately predicting disease risks, which is an intuitive and concise way of presenting model. Therefore, the objectives of the present study were to develop and validate a clinical applicable nomogram for the prediction of POD in TBAD patients who underwent endovascular treatment.

## Methods

### Study design

This was a single-center retrospective cohort study. Ethical approval was obtained from the institutional review board for this study, and the requirement for informed consent was waived. A nomogram-based prediction model of POD was first developed, and then internal and external validations were performed.

### Study population

Adult patients with TBAD who underwent TEVAR were included in this study. The diagnosis of TBAD was confirmed by computed tomography angiography scans. Patients were excluded from the study if they met the following criteria: (1) Marfan syndrome or Ehlers–Danlos syndrome; (2) AD secondary to trauma, iatrogenic injury, or pregnancy; (3) history of malignant tumors; (4) preexisting dementia or cognitive impairment; and (5) presented with preoperative POD.

### Data sources

Data from consecutive TBAD patients between 1 January 2019 and 31 July 2021 were collected at Tongji Hospital affiliated to Tongji Medical College of Huazhong University of Science / Technology, which is a tertiary academic hospital with rich resource of AD cases. The data were split by time into two groups, which was considered a stronger design than randomly split according to the “Transparent Reporting of a multivariable prediction model for Individual Prognosis or Diagnosis (TRIPOD) statement” (17). The development cohort included the data from the first 24 months of the period (1 January 2019 to 31 December 2020) to develop the prediction model. The validation cohort included the data from the last 7 months of the period (1 January 2021 to 31 July 2021) to externally validate the predictive performance of the developed prediction model.

### Outcome definition

Given the aim of the present study, the main outcome measure was the first-time development of POD within 30 days after TEVAR intervention among TBAD patients. POD was determined from the medical chart review method validated in previous studies (18–20): symptom records from daily medical and nursing documents were checked, and the POD may be recorded as “Delirium, confusion, sundowning, somnolent, crying out, inattentive, disorientation, incoherent, hallucinating, restlessness, combative, metabolic encephalopathy, acute confusional state, acute organic mental disorder, or acute organic brain syndrome” (18). Specifically, daily chart review during the ICU and general ward stay was conducted to detect evidence of POD. A transient confusion in anesthesia recovery room was not classified into POD group.

### Potential predictors

A comprehensive literature review was performed to identify potential predictive factors of POD among TBAD patients and guide data extraction. Demographic characteristics, comorbidity, dissection-specific features, clinical symptoms and signs, management and treatment, postoperative laboratory and imaging findings, and sedatives use were collected using standardized data forms. Demographic variables collected for the study included age, sex, and body mass index (BMI). Comorbidity included hypertension, atherosclerosis, diabetes mellitus, stroke, prior cardiac surgery, coronary artery disease, and chronic lung disease. The Charlson Comorbidity Index (CCI) was calculated to define the presence of overall comorbidity burden. Symptoms and signs included initial presentation with syncope or coma, sharp pain, chest pain, postoperative fever, and postoperative hypotension. Dissection-specific features included time from symptoms to surgery and acute AD. Acute AD was defined as the time from symptoms to surgery <14 days (5). Management and treatment factors included anesthetic regimes, duration of surgery, postoperative blood transfusion, and mechanical ventilation. Postoperative imaging findings included pulmonary infection, pleural effusion and atelectasis. Postoperative laboratory findings included estimated glomerular filtration rate (eGFR), serum sodium, serum calcium, serum potassium, albumin, and neutrophil to lymphocyte ratio. For laboratory findings, only the first measurements made postoperatively were collected. Postoperative sedative use included benzodiazepine and analgesics (morphine, fentanyl, or pethidine). All postoperative variables were events that occurred or test results obtained after surgery but before delirium.

### Statistical analysis

The minimum required sample size for development of the prediction model was calculated according to the criteria proposed by Riley et al. (21), at least 10 outcome events are needed per variable (EPV ≥ 10). A POD incidence of 13.3% (11) and a C statistic of 0.80 were used to estimate the sample size. To allow 7 or fewer predictors in the final multivariable model, we estimated that at least 421 patients were needed for the development cohort using the “*pmsampsize*” package in R. Patients with missing data were excluded, and no imputation was performed.

### Characteristics comparison between the delirium and non-delirium groups

Summary statistics are presented using the mean ± standard deviation (SD) or the median and interquartile range percentile (IQR) for continuous variables and frequency counts and percentages for categorical variables. In comparisons between groups, Student's t test or the Mann-Whitney U test was used for continuous variables, depending on the data distribution. The chi-square test or Fisher’s exact test was used for categorical variables as appropriate.

### Variable selection and development of a prediction model

A combination of information on prior known risk factors and clinical judgement was used to identify potential variables (Table 1). Among these, 15 candidate variables with a *p* value near or less than 0.05 in the group comparison were selected, and logistic regression analyses were conducted to determine the associations between these candidate variables and POD. First, a univariable logistic regression model was fit for each candidate variable. The odds ratio (OR) estimates and 95% confidence intervals (CIs) were computed with a value of *p* < 0.05 considered significant. To identify independent predictors of POD, variables with a *p* < 0.05 in the univariable analysis were then entered into the multivariable model using a backward stepwise selection procedure based on the Akaike information criterion. Only independent risk factors (*p* < 0.05) remained in the final model.

**Table 1:** Demographics and clinical characteristics among TBAD patients in the development cohort.

Variables	Total (*n* = 434)	No Delirium (*n* = 369)	Delirium (*n* = 65)	*p*-value
Demographic characteristics				
Age, years, Mean ± SD	57.5 ± 11.3	56.9 ± 11.3	60.7 ± 10.9	0.011*
Age ≥60 years	186 (42.9)	149 (40.4)	37 (56.9)	0.013*
Female, *n* (%)	49 (11.3)	41 (11.1)	8 (12.3)	0.779
BMI, kg/m^2^, Mean ± SD	25.0 ± 3.7	25.0 ± 3.7	25.1 ± 3.9	0.828
Comorbidity				
Hypertension, *n* (%)	372 (85.7)	319 (86.4)	53 (81.5)	0.297
Atherosclerosis, *n* (%)	87 (20.0)	77 (20.9)	10 (15.4)	0.309
Diabetes mellitus, *n* (%)	24 (5.5)	20 (5.4)	4 (6.2)	1.000
Stroke, *n* (%)	30 (6.9)	22 (6.0)	8 (12.3)	0.111
Prior cardiac surgery, *n* (%)	30 (6.9)	26 (7.0)	4 (6.2)	1.000
Coronary artery disease, *n* (%)	30 (6.9)	27 (7.3)	3 (4.6)	0.598
Chronic lung disease, *n* (%)	22 (5.1)	19 (5.1)	3 (4.6)	1.000
CCI ≥ 3, *n* (%)	125 (28.8)	98 (26.6)	27 (41.5)	0.014*
Symptoms and signs				
Syncope or coma, *n* (%)	36 (8.3)	15 (4.1)	21 (32.3)	<0.001*
Sharp pain, *n* (%)	183 (42.2)	153 (41.5)	30 (46.2)	0.480
Chest pain, *n* (%)	282 (65.0)	236 (64)	46 (70.8)	0.288
Postoperative fever, *n* (%)	206 (47.5)	168 (45.5)	38 (58.5)	0.054
Postoperative hypotension, *n* (%)	74 (17.1)	58 (15.7)	16 (24.6)	0.079
Dissection-specific features				
Time from symptoms to surgery, day, Median (IQR)	2.1 (1.2–4.1)	2.2 (1.2–4.1)	1.9 (1.2–3.2)	0.173
Acute AD, *n* (%)	400 (92.2)	337 (91.3)	63 (96.9)	0.122
Management				
General anesthesia, *n* (%)	252 (58.1)	207 (56.1)	45 (69.2)	0.048*
Duration of surgery, hours, Median (IQR)	1.7 (1.0–2.3)	1.7 (1.0–2.3)	2.0 (1.4–2.3)	0.016*
Postoperative blood transfusion, *n* (%)	33 (7.6)	19 (5.1)	14 (21.5)	<0.001*
Postoperative imaging				
Pulmonary infection, *n* (%)	41 (9.4)	29 (7.9)	12 (18.5)	0.007*
Pleural effusion, *n* (%)	99 (22.8)	74 (20.1)	25 (38.5)	0.001*
Atelectasis, *n* (%)	83 (19.1)	59 (16.0)	24 (36.9)	<0.001*
Postoperative Laboratory tests				
eGFR < 8*0* ml/min/1.73 m^2^, *n* (%)	226 (52.1)	182 (49.3)	44 (67.7)	0.006*
Serum sodium <135 mmol/L, *n* (%)	47 (10.8)	40 (10.8)	7 (10.8)	0.986
Serum calcium <2.1 mmol/L, *n* (%)	132 (30.4)	102 (27.6)	30 (46.2)	0.003*
Serum potassium <3.5 mmol/L, *n* (%)	63 (14.5)	57 (15.4)	6 (9.2)	0.190
Albumin <30 g/L, *n* (%)	30 (6.9)	18 (4.9)	12 (18.5)	<0.001*
Neutrophil to lymphocyte ratio, Median (IQR)	9.7 (6.4–14.9)	9.6 (6.3–14.3)	10.5 (7.7–22.5)	0.011*
Postoperative sedatives use				
Benzodiazepines, *n* (%)	45 (10.4)	32 (8.7)	13 (20.0)	0.006*
Analgesics, *n* (%)	48 (11.1)	43 (11.7)	5 (7.7)	0.348

Abbreviations: TBAD, type B aortic dissection; BMI, body mass index; CCI, the Charlson Comorbidity Index; eGFR, estimated glomerular filtration rate; SD, standard deviation; IQR, interquartile range percentile.

*means *p*-value <0.05.

To validate the logistic regression analysis results, the least absolute shrinkage and selection operator (LASSO) method using 10-fold cross-validation was also applied to select the most useful predictive variables. In total, 15 potential predictors were included in the selection process. The most predictive variables were selected using one standard error of the minimum lambda (lambda.1se).

To obtain a better application in a clinical setting, a nomogram was built based on the final multivariable logistic regression model in the development cohort. Each regression coefficient was proportionally converted into a 0-point to 100-point scale.

### Assessment and validation of the POD prediction nomogram

The receiver operating characteristic (ROC) curve was plotted, and the area under the ROC curve (AUC) was used to assess the overall discriminative ability of the final prediction model. Model calibration was measured by calculating the Brier score, which is the mean square difference between predictive probability and observed outcome (22). Scaled from 0 to 1, a lower Brier score indicates better performance of the model. Model calibration was also assessed using the Hosmer–Lemeshow goodness-of-fit test. A significant *p* value (<0.05) of the test indicated poor calibration.

To adjust for over-optimism in the estimation of model performance, an internal validation of the accuracy estimates was conducted using the bootstrap technic with 100 resamples to calculate the adjusted AUC and Brier score.

External validation was carried out independently following the development of the nomogram and using data from the validation cohort. The risk for each patient was calculated by the developed nomogram, and then its predictive performance of discrimination and calibration was assessed using the AUC, Brier score and Hosmer–Lemeshow test statistic.

Statistical analysis was conducted with SPSS (version 25.0) and R software (version 4.1.2). The packages in R software that were mainly used in this study were “glmnet”, “rms” and “pROC”.

## Results

### Patient characteristics of the development cohort

**Figure 1:**
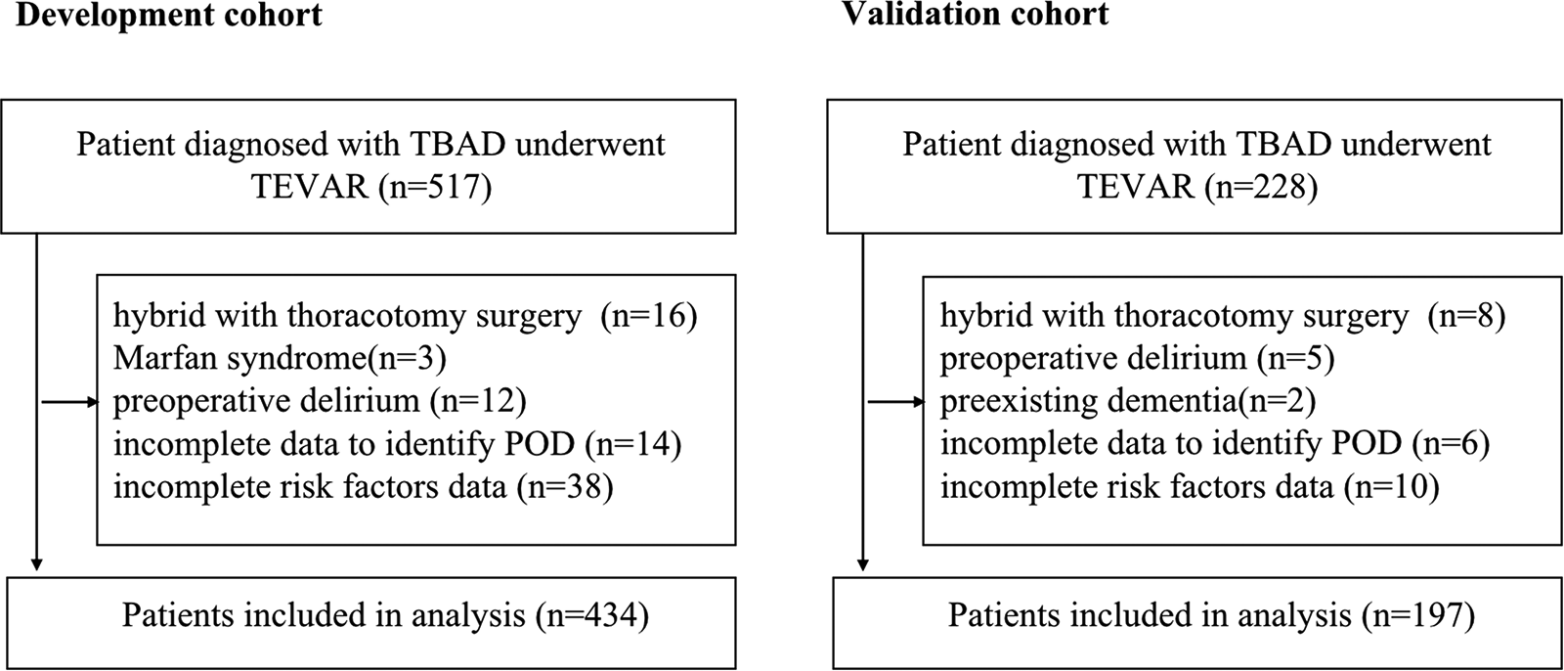
Flow chart of the selection of patients.

A total of 517 TBAD patients were identified in the development cohort, of whom only a small number of patients received TEVAR combined with thoracotomy aortic surgery, were diagnosed with Marfan syndrome or had preoperative delirium, and these were excluded to reduce the heterogeneity. In the end, 434 patients with adequate data were included for the final analysis (Figure 1). A total of 65 patients developed POD (15.0%). The mean age was 57.5 ± 11.3 years, and 42.9% (186 patients) were older than 60 years. There were only 49 female patients (11.3%) in this cohort. A major proportion of the patients had comorbid hypertension (85.7%). Thirty patients (6.9%) had prior cardiac surgery. For the condition of multimorbidity, more than a quarter of the patients had a CCI score ≥3. Detailed characteristics are summarized in Table 1.

### Predictor selection and construction of the POD-predicting nomogram

The results from both univariable and multivariable logistic regression analyses were provided in Table 2. Based on the results of comparison between the delirium and non-delirium groups in Table 1, 15 candidate variables for POD were investigated by performing a univariate logistic regression analysis and 13 variables were found to be statistically significant (*p* < 0.05). Thereafter, the 13 predictors were entered in a multivariable logistic regression analysis (backward stepwise method), which indicated that 7 were independent predictors of POD in TBAD patients, including age≥ 60 years (OR, 2.31; 95% CI, 1.23–4.47; *p *= 0.011), syncope or coma (OR, 14.49; 95% CI, 6.13–35.84; *p *< 0.001), postoperative blood transfusion (OR, 3.54; 95% CI, 1.42–8.53; *p *= 0.005), atelectasis (OR, 2.50; 95% CI, 1.23–4.96; *p *= 0.010), eGFR < 80 ml/min/1.73 m^2^ (OR, 2.06; 95% CI, 1.08–4.04; *p *= 0.030), albumin< 30 g/L (OR, 3.19; 95% CI, 1.22–8.05; *p *= 0.015), and neutrophil to lymphocyte ratio (OR, 1.05; 95% CI, 1.01–1.08; *p *= 0.005).

**Table 2:** Univariable and multivariable logistic regression analysis for candidate variables of POD in TBAD patients underwent TEVAR in the development cohort.

Variables	Univariable analysis			Multivariable analysis**		
	OR	95%CI	*p*-value	OR	95%CI	*p*-value
Demographic and comorbidity						
Age ≥60 years**	1.95	1.14–3.33	0.014*	2.31	1.23–4.47	0.011*
CCI ≥ 3**	1.96	1.14–3.39	0.015*	-	-	-
Symptoms and signs						
Syncope or coma**	11.26	5.41–23.44	<0.001*	14.49	6.13–35.84	<0.001*
Postoperative fever	1.68	0.99–2.87	0.056	-	-	-
Management						
General anesthesia	1.76	1.00–3.10	0.050	-	-	-
Duration of surgery, hours**	1.25	1.02–1.55	0.033*	-	-	-
Postoperative blood transfusion**	5.06	2.39–10.71	<0.001*	3.54	1.42–8.53	0.005*
Postoperative imaging						
Pulmonary infection**	2.65	1.28–5.52	0.009*	-	-	-
Pleural effusion**	2.49	1.42–4.37	0.001*	-	-	-
Atelectasis**	3.08	1.73–5.47	<0.001*	2.50	1.23–4.96	0.010*
Postoperative Laboratory tests						
eGFR <80 ml/min/1.73 m^2^**	2.15	1.23–3.76	0.007*	2.06	1.08–4.04	0.030*
Serum calcium <2.1 mmol/l**	2.24	1.31–3.84	0.003*	-	-	-
Albumin <30 g/L**	4.42	2.01–9.68	<0.001*	3.19	1.22–8.05	0.015*
Neutrophil to lymphocyte ratio**	1.05	1.02–1.08	<0.001*	1.05	1.01–1.08	0.005*
Postoperative sedatives use						
Benzodiazepines**	2.63	1.30–5.34	0.007*	-	-	-

Note: - Not available.

* *p*-value  < 0.05.

** All the 13 variables with a *p* < 0.05 in univariate logistic regression analysis were entered in a multivariable logistic regression analysis (backward stepwise), resulting 7 independent predictors of POD.

Abbreviations: TBAD, type B aortic dissection; POD, postoperative delirium; TEVAR, thoracic endovascular aortic repair; CCI, the Charlson Comorbidity Index; eGFR, estimated glomerular filtration rate.

**Figure 2:**
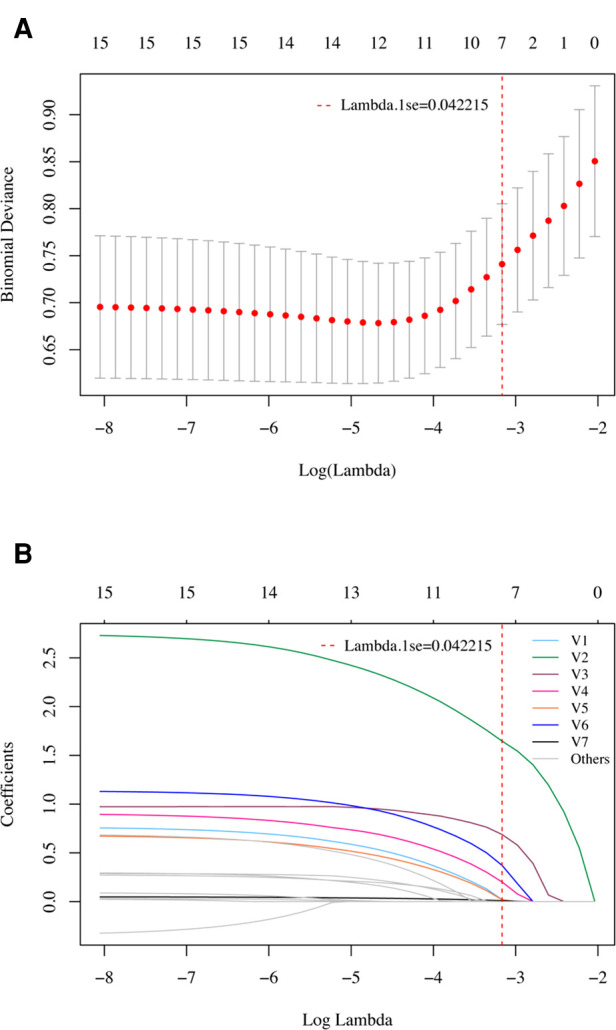
Variable selection using the LASSO regression model in the development cohort. (**A**) Tuning parameter (lambda) selection in the LASSO model using 10-fold cross-validation. A vertical line was drawn at the optimal values by using the 1 standard error of the minimum criteria (the 1 se criteria). (**B**) LASSO coefficient profiles of the 15 potential variables. A vertical line was drawn at the value selected with 10-fold cross validation, where optimal lambda (lambda.1se) resulted in 7 variables with nonzero coefficients. V1, Age ≥ 60 years; V2, Syncope or coma; V3, Postoperative blood transfusion; V4, Atelectasis; V5, eGFR < 80 ml/min/1.73 m^2^; V6, Albumin < 30 g/L; V7, Neutrophil to lymphocyte ratio. These variables were fully consistent with the 7 independent predictors selected by univariate and multivariate logistic regression models.

In the LASSO regression model, the 15 candidate variables were reduced to the 7 most predictive variables with nonzero coefficients using the “lambda.1se” criteria, including age ≥ 60 years, syncope or coma, postoperative blood transfusion, atelectasis, eGFR <80 ml/min/1.73 m^2^, albumin <30 g/L, and neutrophil to lymphocyte ratio (Figure 2). This result is fully consistent with independent predictors selected by the logistic regression model, as stated.

**Figure 3:**
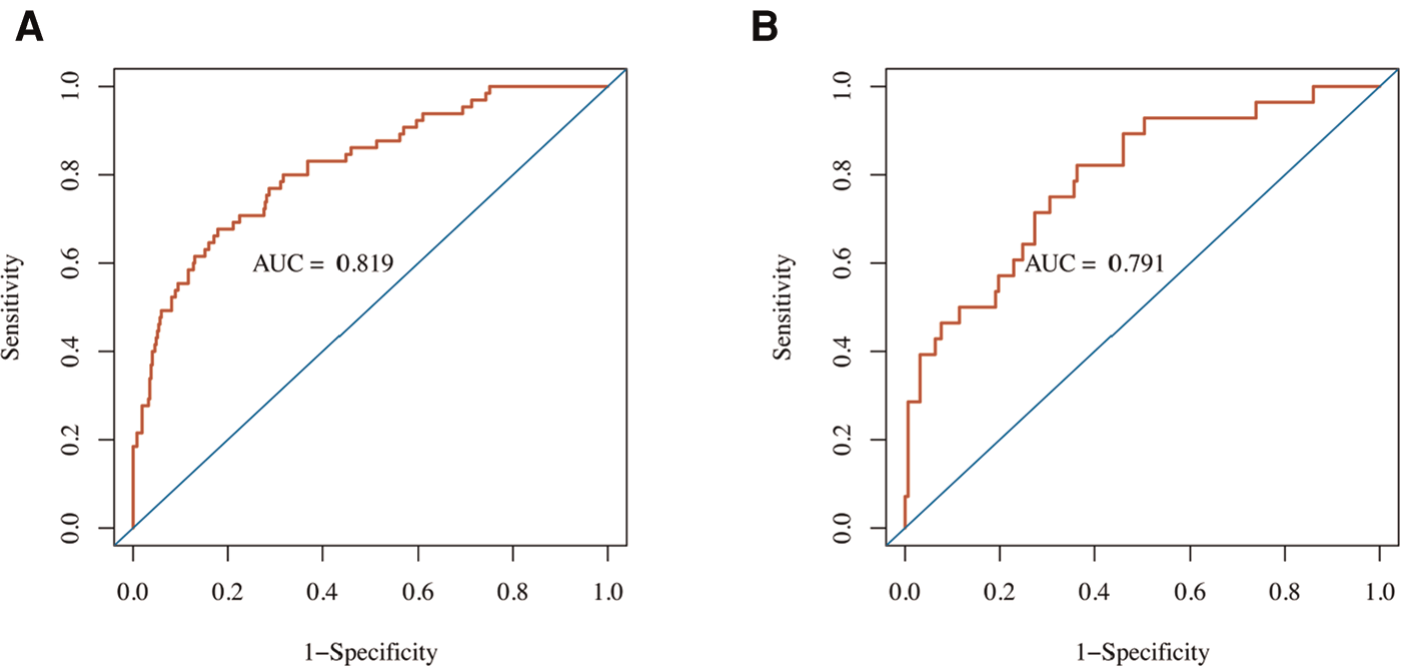
Performance of the nomogram predicting POD for TBAD patients who underwent TEVAR. ROC curves showed AUCs for the nomogram of POD prediction in the (**A**) development cohort and (**B**) validation cohort. AUC, area under the ROC curve; ROC, receiver operating characteristic.

**Figure 4:**
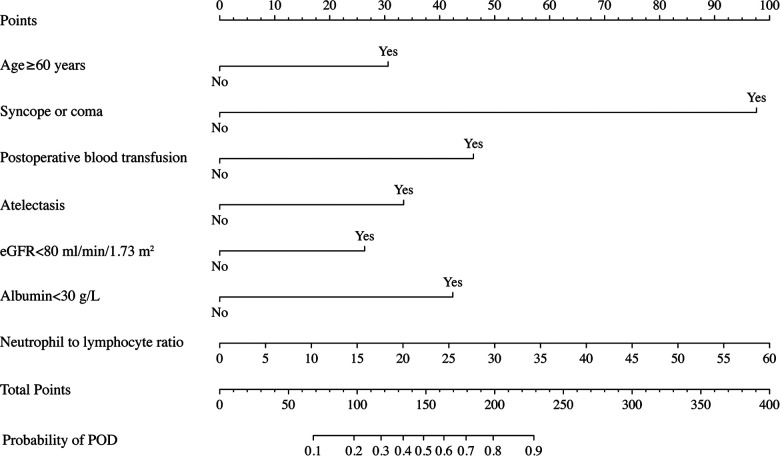
Nomogram to predict POD probability for TBAD patients who underwent TEVAR on the basis of multivariable logistic analysis results.

The final prediction model consisting of 7 risk predictors was then constructed, and the nomogram was developed based on the weight of each predictor (Figure 4). Every predictor can obtain a score according to the top point line (0–100), and the total scores across factors are converted to the POD probability of TBAD patients who underwent TEVAR.

### Performance of the POD-predicting nomogram

The nomogram demonstrated good discriminative ability with an AUC of 0.819 (95% CI, 0.762–0.876) in the development cohort (Figure 3). A Brier score of 0.095 and no statistically significant Hosmer–Lemeshow statistic (*p *= 0.492 > 0.05) both indicated good calibration of the nomogram (Table 3).

**Table 3:** Performance of the POD-predicting nomogram.

	AUC (95% CI)	Brier score	Hosmer– Lemeshow test		
			*χ* ^2^	*df*	*P*
Development cohort	0.819 (0.762–0.876)	0.095	7.423	8	0.492
Internal bootstrap validation	0.797 (0.735–0.849)	0.102	-	-	-
External validation	0.791 (0.700–0.881)	0.095	7.136	8	0.522

Abbreviations: AUC, area under the ROC curve; df, degree of freedom; χ^2^, Chi-Square.

### Internal validation

By internal bootstrap validation, the optimism-adjusted AUC of the nomogram was 0.797 (95% CI, 0.735–0.849). The optimism-adjusted Brier score was 0.102, which was similar to that in the primary development dataset. The bootstrap validation analysis showed that the model was stable and reliable.

### External validation

In the external (temporal) validation cohort, 197 patients with sufficient data were included for the final analysis (Figure 1). Demographics and clinical characteristics of patients in validation cohort was shown in Table 4. A total of 28 patients developed POD (14.2%). Good discriminative ability of the nomogram for POD prediction was also observed in the validation cohort (Figure 3), with an AUC of 0.791 (95% CI, 0.700–0.881). Furthermore, the favorable calibration of the nomogram was confirmed in the validation cohort, with a Brier score of 0.095 and a nonsignificant Hosmer–Lemeshow test statistic (*p* = 0.522 > 0.05). The results demonstrated that the nomogram has reasonable predictive ability against an external (temporal) dataset.

**Table 4:** Demographics and clinical characteristics among TBAD patients in the validation cohort.

Variables	Total (*n* = 197)	No Delirium (*n* = 169)	Delirium (*n* = 28)
Demographic characteristics			
Age, years, Mean ± SD	56.2 ± 10.5	56.0 ± 10.5	57.3 ± 11.1
Age ≥60 years	69 (35.0)	57 (33.7)	12 (42.9)
Female, *n* (%)	32 (16.2)	26 (15.4)	6 (21.4)
BMI, kg/m^2^, Mean ± SD	25.3 ± 3.6	25.4 ± 3.5	24.6 ± 4.3
Comorbidity			
Hypertension, *n* (%)	177 (89.8)	153 (90.5)	24 (85.7)
Atherosclerosis, *n* (%)	52 (26.4)	46 (27.2)	6 (21.4)
Diabetes mellitus, *n* (%)	11 (5.6)	10 (5.9)	1 (3.6)
Stroke, *n* (%)	6 (3.0)	5 (3.0)	1 (3.6)
Prior cardiac surgery, *n* (%)	15 (7.6)	13 (7.7)	2 (7.1)
CCI ≥ 3, *n* (%)	54 (27.4)	47 (27.8)	7 (25.0)
Other predictors			
Syncope or coma, *n* (%)	15 (7.6)	5 (3.0)	10 (35.7)
Postoperative blood transfusion, *n* (%)	33 (7.6)	19 (5.1)	14 (21.5)
Atelectasis, *n* (%)	83 (19.1)	59 (16.0)	24 (36.9)
eGFR < 80 ml/min/1.73 m^2^, *n* (%)	226 (52.1)	182 (49.3)	44 (67.7)
Albumin <30 g/L, *n* (%)	30 (6.9)	18 (4.9)	12 (18.5)
Neutrophil to lymphocyte ratio, Median (IQR)	9.7 (6.4–14.9)	9.6 (6.3–14.3)	10.5 (7.7–22.5)

Abbreviations: TBAD, type B aortic dissection; BMI, body mass index; CCI, the Charlson Comorbidity Index; eGFR, estimated glomerular filtration rate; SD, standard deviation; IQR, interquartile range percentile.

## Discussion

The occurrence rates of POD observed in the development and validation cohorts were approximately 15.0% and 14.2%, which are comparable to those in a previous investigation with a similar population in China (11). This rate is lower than rates reported in other studies among patients with TAAD (12, 23, 24), which might be attributable to varying degrees of arch vessel involvement. Evidence has established that POD could prominently result in longer hospital stays and ICU stays, increase the use of hospital resources, and affect early and follow-up mortality among these patients (11). Given the adverse consequences, the prediction of delirium after TEVAR is clearly clinically relevant.

To our knowledge, this is the first delirium prediction study for POD in a cohort of TBAD patients after TEVAR. The final selected predictors were double verified by logistic regression and LASSO regression, indicating that the results were robust. We developed a prediction model presented in the form of a simple nomogram for POD probability and performed preliminary external validation, with both the development and validation cohorts showing good discriminant power (AUCs, 0.819 and 0.791, respectively). A nomogram is an advanced statistical method that can incorporate multiple factors into the prediction and consider the weight of each variable, allowing the calculation of risk estimates for each individual patient, which is beneficial to clinical practice. The nomogram in this study has the potential to enable caregivers to achieve risk stratification based on local conditions and may then assist in their decision making of preventive measures. Future trials can use the nomogram to categorize patients into low-, moderate-, and high-risk groups for developing delirium early and then investigate different preventive interventions to improve care efficiency and quality while increasing cost-effectiveness (25).

Risk factors contributing to the predictive model included age ≥60 years, presentation with syncope or coma, postoperative blood transfusion, atelectasis, eGFR < 80 ml/min/1.73 m^2^, albumin <30 g/L, and neutrophil to lymphocyte ratio. These seven variables were generally readily available in clinical settings, indicating that the model is of high value in daily practice. Some were basically consistent with previously published risk factors for delirium. For example, increasing age has been widely reported as the most consensual nonmodifiable risk factor in many studies (9, 15, 26, 27), which was again confirmed in the present study. However, specifically, ≥65 years is outlined in some guidelines as an important risk factor for POD (28, 29). It was found in the present study that age ≥60 years already significantly increases the risk of developing POD. This inconsistency may be related to the specific disease characteristics and epidemiological features of AD. Our study enriches the existing literature on POD in AD patients. In addition, patients with worse renal function or lower albumin are more likely to suffer from delirium, which has also been commonly reported in previous observations (11, 12, 30). Moreover, inflammation has been reported to play a critical role in the development of delirium (31). To improve clinical utility of our nomogram, only routinely measured inflammatory markers with relatively low costs were taking into consideration, rather than C-reactive protein, interleukin (IL)-6, IL-8, et al. The neutrophil to lymphocyte ratio is a more comprehensive biomarker of the rapid response to the degree of inflammation progression, which has increasingly drawn the attention of many researchers (32). In the present study, an elevated neutrophil to lymphocyte ratio can independently predict POD among AD patients, as hypothesized. This result is in accord with recent findings in older internal medicine patients (16).

According to the 2018 Clinical Practice Guidelines, blood transfusion administration is a modifiable risk factor with strong evidence for an association with delirium in critically ill adults (33). The present study demonstrated the risk of postoperative blood transfusion for delirium. The possible reason is that anemia or blood loss is highly correlated with transfusion, which may cause reduced cerebral blood flow and impair tissue oxygenation capacity, thereby causing delirium (34, 35). In addition, blood transfusion itself may cause POD since it may result in dysregulation of cytokines and further amplify the systemic inflammatory response (34). Further studies are needed to confirm the role of blood transfusion in the development of POD. Atelectasis could contribute to hypoxemia, which is an underlying cause of delirium (36). Early detection and initiation of appropriate treatment is useful for delirium prevention. For sedatives, although univariate analysis showed that benzodiazepines were a significant risk factor for POD (OR = 2.63, *p* = 0.007, Table 2). This association was no longer significant after adjustment of confounding factors. However, previous evidence suggested that administration of benzodiazepines is associated with an increased risk of delirium, and avoiding routine benzodiazepine use is recommended by current guidelines (28, 30, 33, 37).

Of importance, the present study has the novel addition of presentation symptoms to obtain a more comprehensive delirium risk evaluation. It is somewhat surprising that none of the previous studies on delirium in AD patients included clinical manifestations. In fact, according to observational results from the International Registry of Acute Aortic Dissection (IRAD) study (38, 39), clinical presentation, especially nervous system symptoms, seems to have an important connection with neurological complications and outcomes. Bossone and colleagues (38) found that syncope on presentation was 3-fold higher among acute TAAD patients with stroke. Similar to their results, presented with syncope or coma is a significant independent factor for predicting delirium after TEVAR in the TBAD population. These findings are important complementary to the literature. Further studies are warranted to investigate the specific relationship between these symptoms and POD and elucidate the detailed mechanism.

### Limitation

There are certain limitations that must be considered, and the current results should be interpreted with caution. First, although the present study was adequately powered, the sample size is relatively modest. Thus, further studies are needed to verify the conclusion of the study. Second, this was a single-center retrospective study that lacked prospective data of POD assessment; the possibility of a false negative may exist since delirium is a fluctuating condition. And the exclusion of patients lacking important data may also introduce bias. Future external validation of the model based on prospective data from other institutions may help further test the predictive power of the nomogram and improve its generalizability. Third, though initial presented with syncope or coma was found to be association with POD in the TBAD population, we were unable to investigate whether such patients developed cerebrovascular events due to insufficient cerebral imaging data. Further explorations in a prospective fashion are recommended to clarify this issue. Finally, the selection of risk factors may also not sufficiently comprehensive. Dissection-specific variables, such as complicated or stable dissection, anatomic features, and procedure details, should thus be explored in future studies to improve performance of the prediction model.

## Conclusion

This study developed and validated a POD prediction nomogram for TBAD patients after TEVAR that revealed sufficient validity. It enables healthcare providers to effectively implement risk identification of POD using only seven predictors. These findings may provide a basis for improved risk stratification and refinements to POD preventive intervention among this population.

## Data Availability

The datasets used and/or analyzed during the current study are available from the corresponding author on reasonable request.
